# Characterization of the head-to-trunk orientation with handheld optical 3D apparatus based on the fringe projection technique

**DOI:** 10.1186/1475-925X-12-96

**Published:** 2013-09-26

**Authors:** Urban Pavlovčič, Janez Diaci, Janez Možina, Matija Jezeršek

**Affiliations:** 1 University of Ljubljana, Faculty of Mechanical Engineering, Aškerčeva 6, Ljubljana 1000, Slovenia

**Keywords:** 3D measurements, Head-to-trunk orientation, 3D alignment, Handheld 3D measuring system

## Abstract

**Background:**

Knowing the orientation of the head is important in many fields, including medicine. Many methods and measuring systems exist, but usually they use different markers or sensors attached to the subject’s head for head orientation determination. In certain applications these attachments may represent a burden or a distraction to the subject under study which may have an unfavourable impact on the measurement. We propose a non-contact optical method for head-to-trunk orientation measurement that does not require any attachments to the subject under study.

**Methods:**

An innovative handheld 3D apparatus has been developed for non-invasive and fast 3D shape measurements. It is based on the triangulation principle in combination with fringe projection. The shape of the subject’s upper trunk and head is reconstructed from a single image using the Fourier transform profilometry method. Two shape measurements are required to determine the head-to-trunk orientation angles: one in the reference (neutral) position and the other one in the position of interest. The algorithm for the head-to-trunk orientation angle extraction is based on the separate alignment of the shape of the subject’s upper trunk and head against the corresponding shape in the reference pose. Single factor analysis of variance (ANOVA) was used for statistical characterisation of the method precision.

**Results:**

The method and the 3D apparatus were verified *in-vitro* using a mannequin and a reference orientation tracker. The uncertainty of the calculated orientation was 2°. During the *in-vivo* test with a human subject diagnosed with cervical dystonia (aged 60), the repeatability of the measurements was 3°. *In-vitro* and *in-vivo* comparison was done on the basis of an experiment with the mannequin and a healthy male (aged 29). These results show that only the difference between flexion/extension measured angles was statistically significant. The differences between means were less than 1° for all ranges.

**Conclusions:**

The new non-contact method enables the compensation of the movement of the measuring instrument or the subject’s body as a whole, is non-invasive, requires little additional equipment and causes little stress for the subject and operator. We find that it is appropriate for measurements of the head orientation with respect to the trunk for the characterization of the cervical dystonia.

## Background

Knowing the orientation of the head is important in many different fields such as human-computer interfaces, video compression, face recognition systems, biological experiments and medicine [1-3]. Head orientation measurement techniques are mainly based on a combination of different gyroscopes, accelerometers and electronic compasses. One such system is based on a three-axis accelerometer and a resonator gyroscope mounted on a hard hat [4]. The data acquired by the tilt sensor reached an accuracy of 0.63° and the gyroscope reached an accuracy of 0.52°. Another measuring system was composed of four sensors placed on the person and the stationary electromagnetic transmitter of a Fastrack system [5]. Its static orientation accuracy is 0.15° [6]. Another technique uses two inclinometers attached to each arm of a pair of eyeglasses [7]. Yet another uses two intersecting plastic protractors, a grid pattern and a laser mounted on a helmet [8]. The rotations in the sagittal and transverse plane were calculated employing the location of the laser illumination. The rotation in the coronal plane was calculated from protractors. Nintendo Wii remote controllers fixed to a mechanical frame and an IR beacon, mounted on the head were also used to measure the head posture. The standard deviation of 15 repeated measurements ranged from 0.56° to 2.70° [3]. The head orientation can be also determined by using 2D camera. One technique is based on finding the best translation and rotation of the pre-measured 3D shape of the subject’s face according to the acquired 2D image [2]. The reported standard deviation is 2.3 mm in position and 0.47° in orientation. The second 2D camera based technique uses a template tracking algorithm that automatically extracts neck angles from sagittal videos [9].

Although the presented systems achieve high levels of measuring uncertainty, our concerns are related mostly to the fixation of the different markers on the subject’s body. In our opinion it is very hard to ensure that the junction between the marker and the human body is rigid. The systems are also usually quite large and difficult to transport. Other weakness of the above mentioned methods is impossibility to distinguish between the head rotations caused by the neck and the rotations caused by the torso twisting, because the torso rotations are not monitored.

This paper presents a novel method that enables the measurement of the head posture relative to the trunk. The method has been developed with the aim of characterizing the effects of cervical dystonia, a disease which affects the patient’s head posture and the range of movement as well as their quality of life in many other ways [10,11]. The main effects on the head posture are the head lateral tilt in the body’s coronal plane (laterocollis), head rotation in transversal plane (torticollis) and flexion (anterocollis) or extension (retrocollis) in the body’s sagittal plane. Most patients suffer from a combination of two or more effects [5]. The presented method utilizes optical 3D measurements of the upper trunk and head to measure these effects. The relative rotation of the head with respect to the trunk is calculated from the alignment of the trunk and head measurements to the reference one.

## Methods

### Handheld 3D camera

**Figure 1:**
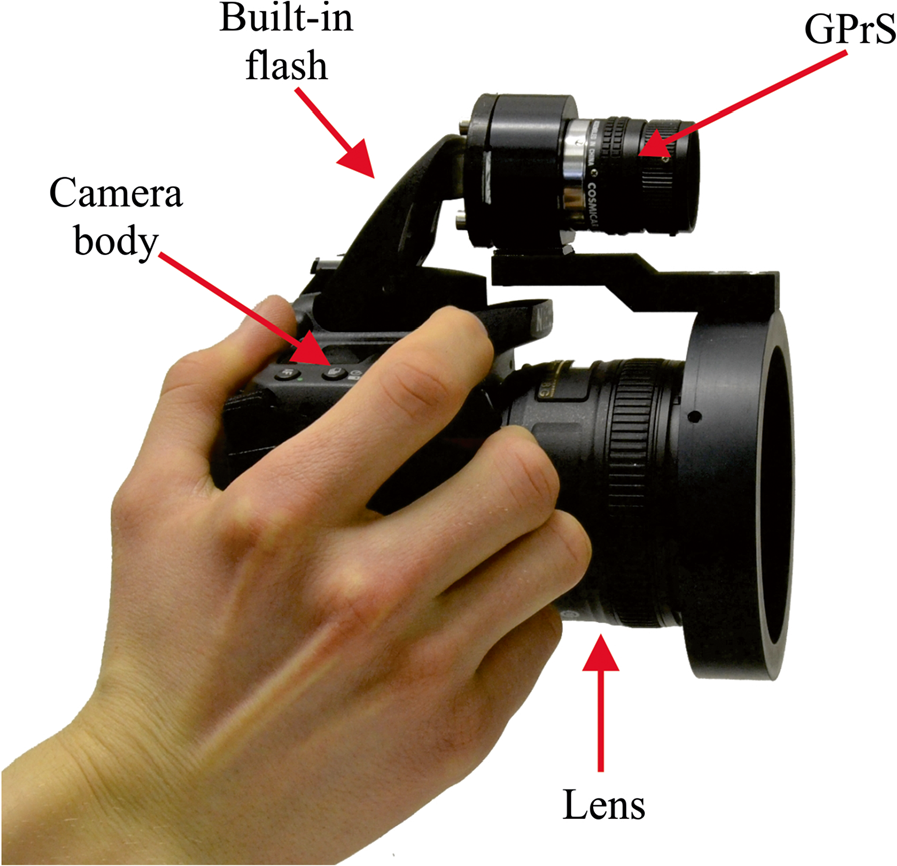
**Handheld 3D camera based on the fringe projection technique.** The instrument is composed of DSLR camera body, fixed focal length lens and grating projection system (GPrS) which uses built-in flash as a light source.

The handheld 3D camera (HHC) is composed of a digital single-lens reflex (DSLR) camera body, lens and grating projection system (GrPS). In our experimental setup, we used a Nikon D90 with a Nikkor 50 mm f/1.8 G AF-S lens. The GrPS is composed of an industrial C-mount lens (Computar M2514-MP, 25 mm, F1.4, 2/3″), a grating mask and a connection arm (see Figure 1). The built-in camera flash was used as a light source. The grating mask was placed on the back of the holder, between the projector lens and the camera flash. The mask is rectangular in shape, approximately 8.8×6.6 mm in size and composed of a 67-stripe Ronchi pattern which was made using a laser micro-marking technique (LPKF MarkLine8V).

**Figure 2:**
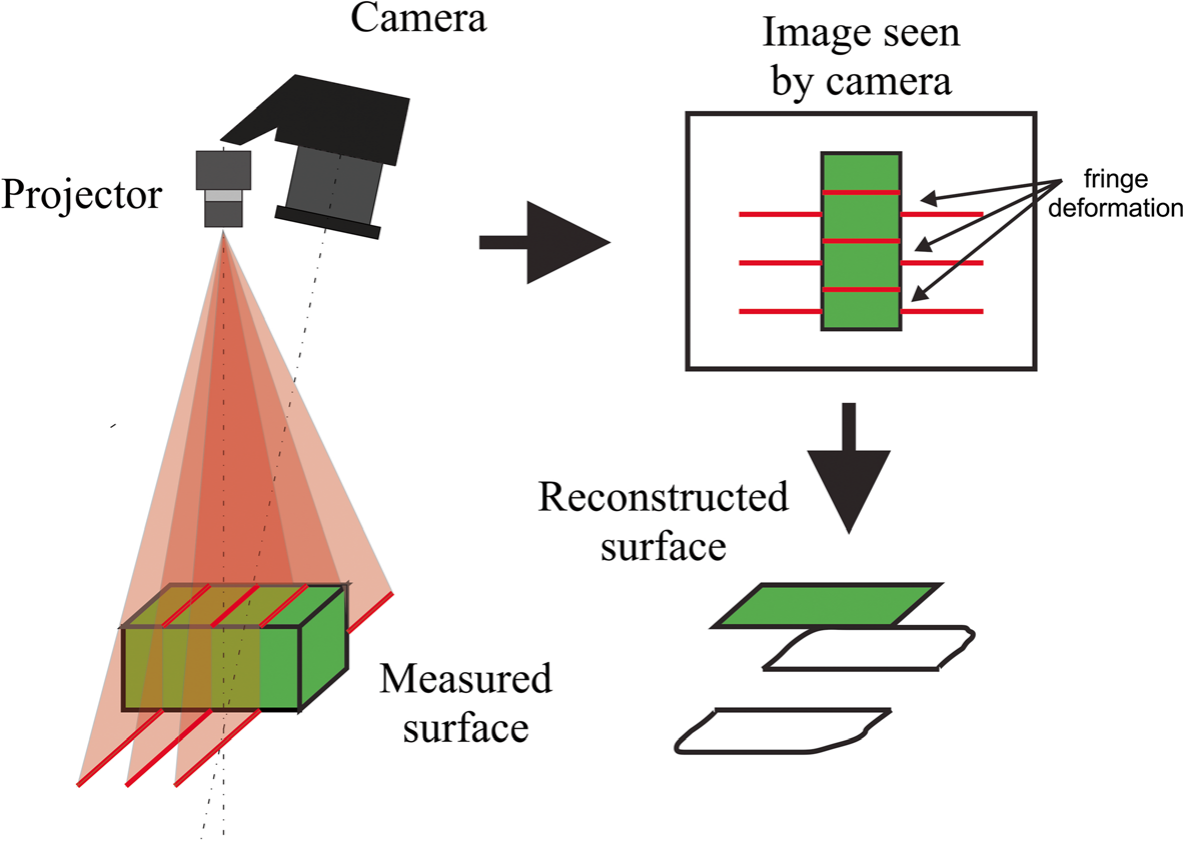
**Schematics of the measuring principle.** The projector illuminates the measured surface by multiple stripes light pattern. The camera captures image from different viewpoint and therefore the stripes on it are deformed according to the geometry of the measured surface which is then reconstructed by the triangulation principle.

For acquiring measurements a picture of the measured surface was captured using the camera flash. The mask was illuminated by the camera flash and the generated light pattern was projected onto the surface by the GrPS lens (see Figure 2). The deformed grating pattern is seen on the captured image. For the surface reconstruction, we transferred the images to a personal computer and processed them with developed surface reconstruction software. It is based on the principle of the Fourier transform profilometry method [12,13], the phase unwrapped by a quality-guided unwrapping algorithm [14] and reconstruction method [15].

The measuring range of the system is 700×520×400 mm at a distance of 2 m. Calibration was done by the reference surface [16]. Ten measurements of the reference surface (plate with semi-circular grooves, of which the dimensions are exactly known) were captured across the measuring range and imported into the software for the optimization of the surface reconstruction parameters. These parameters include the focal length of the lens, the rotations of the camera and the projector, the triangulation angle, the angular separation between the light planes, the camera and lens distortion, the camera sensor dimensions and the image center location. The parameters were numerically optimized until the minimum standard deviation of the displacements between the measured and reference surface was found. After the calibration the standard deviation of the displacement was 1.6 mm.

**Figure 3:**
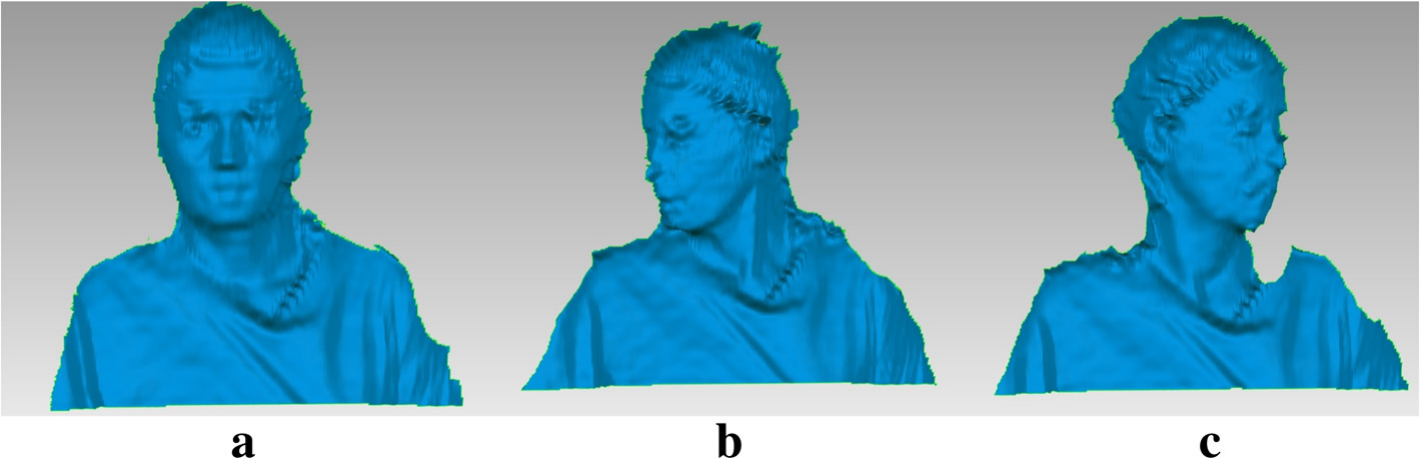
**Examples of measured body shapes.** Examples of measured body shapes. **a)** Subject in the reference position. **b)** Rotation of the head to the right. **c)** Rotation of the head to the left.

In Figure 3 examples of the three reconstructed surfaces of the same human subject, captured by the presented 3D measuring system are shown. The surface seen in Figure 3a was acquired in the reference position and is later aligned in the coordinate system; measurements in Figure 3b and Figure 3c, are registered following the procedure presented in the next section.

### Head-to-trunk rotation extraction from 3D measurements

**Figure 4:**
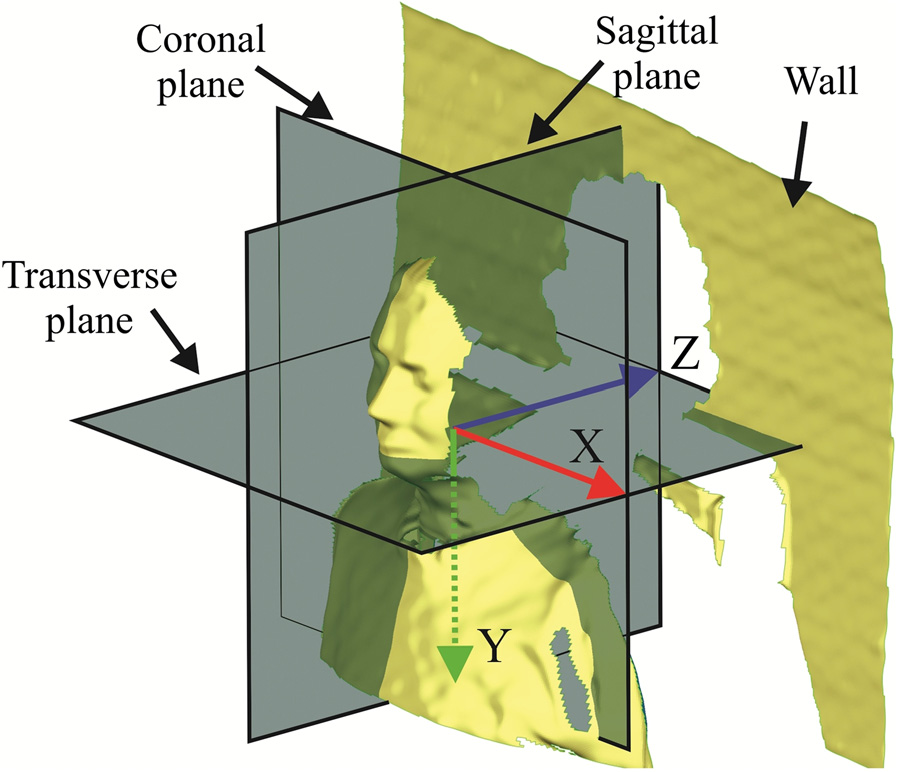
**Example of the reference measurement aligned to the coordinate system.** Example of the reference measurement aligned to the coordinate system with visible body planes.

The rotations of the human body and head can be described as rotations in three body planes; the sagittal, coronal and transverse plane (see Figure 4). Because the software can measure only the rotations in the software’s coordinate system, it is essential that the body planes are parallel to the coordinate system planes of the software. The sagittal body plane is found using the mirroring and aligning procedure [17]. We determined the coronal plane assuming that the body’s coronal plane is parallel to the wall the person was leaning on. The wall was measured at the same time as the person and can be seen in Figure 4. The directions of the X, Y and Z axis are also seen in Figure 4. Any translation of the coronal plane in the Z direction and the transverse plane in the Y direction has no effect on the calculated Euler angles.

Hereinafter, the flexion and extension in the sagittal plane (around the X axis) will be noted as the positive and negative *φ* angle, respectively; the right and left rotation in the transverse plane (around the Y axis) as the positive and negative angles *θ*, respectively, and the left and right lateral tilt (around the Z axis) as the positive and negative angles *ψ*, respectively.

**Figure 5:**
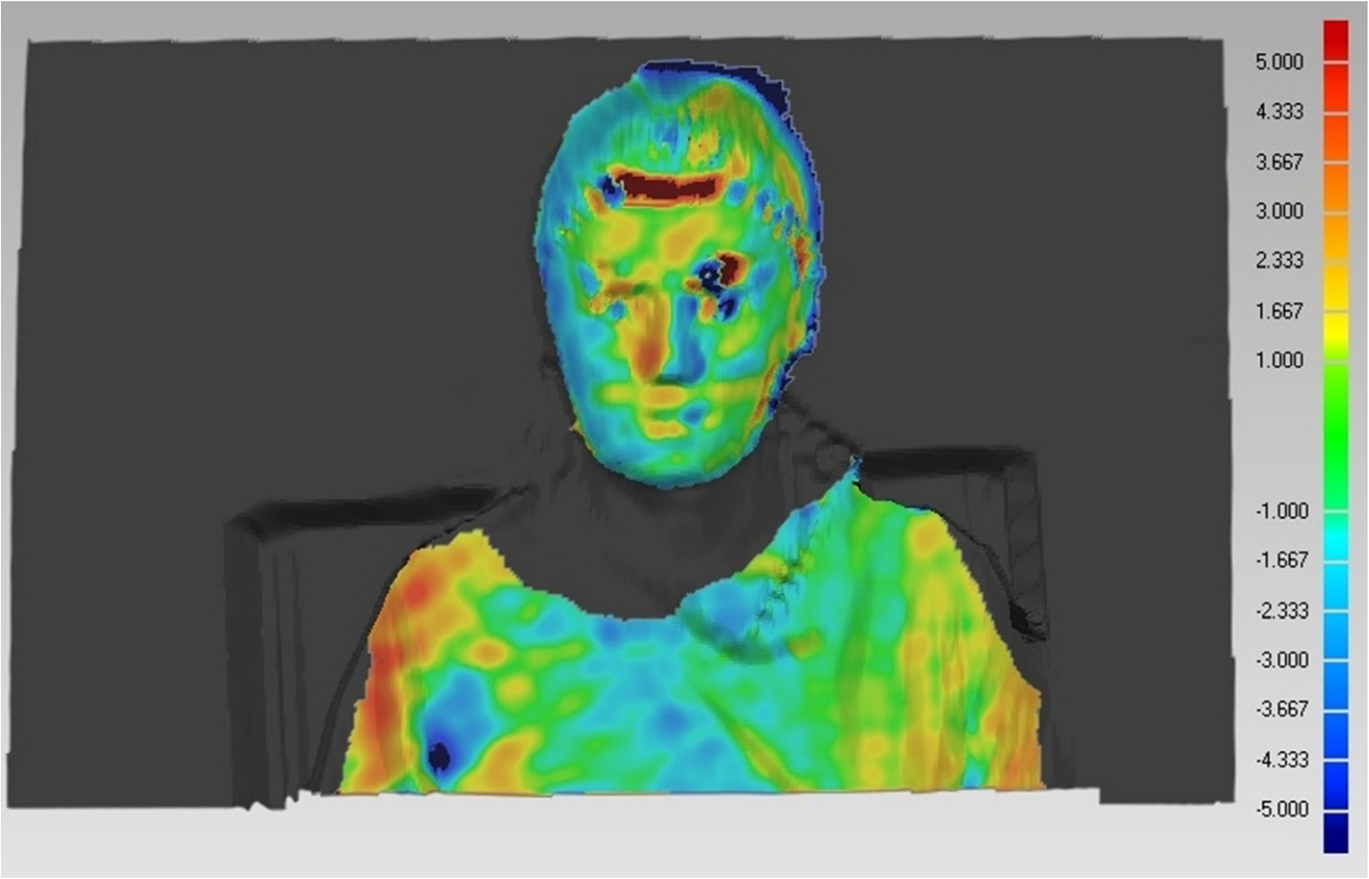
**Example of the deviations after the registration.** Scale on the right is in millimetres.

Each measured surface was split on the subject’s head surface and the subject’s trunk surface, where only the parts of the head or trunk on which we expected a small degree of deformation during the movement are kept for further analysis. This means that the neck and upper arm parts are excluded from the head and trunk partial measurements to enable a reliable determination of the head orientation. The divided measurements are then aligned to the reference measurement by using the Geomagic Studio software (GMS) [18]. An example of such alignment procedure is shown in Figure 5, where the colours indicate the deviations of the currently analysed body position with respect to the reference measurement (shown in grey).

In this manner we obtained a pair of rotation matrices from the GMS for each measurement. We denoted the matrix of the head rotation **R**_*h*_ and matrix of the trunk rotation **R**_*t*_. Due to the possibility of the person moving between consecutive measurements and the movement of the measuring instrument, we calculated the relative head rotation with respect to the trunk **R** as:

(1) $$R=R_{h}\cdot R_{t}^{-1}$$

Since the matrix **R**_*t*_ is orthogonal, the inverse operation can be substituted with transposition, to reduce the computational demands.

The Euler angles, which represent the head-to-torso orientation, are then calculated by the rotation matrix decomposition technique as follows [19]:

(2) $$\varphi =\text{arctan}\left(\frac{-R_{2,3}}{R_{3,3}}\right)$$

(3) $$\theta =\text{arctan}\left(\frac{R_{1,3}}{\sqrt{R_{1,2}^{2}+R_{1,1}^{2}}}\right)$$

(4) $$\psi =\text{arctan}\left(\frac{-R_{1,2}}{R_{1,1}}\right)$$

### *In-vitro* verification

The uncertainty of the measuring method was verified by measuring the upper part of a female mannequin with a movable head. An inertial measurement sensor (Xsens 3DOF orientation Tracker, hereinafter referred to as “XOT”, [20]) was attached to the top of the mannequin’s head to perform reference measurements of the head’s orientation. Since the trunk of the mannequin was immovably fixed, the changes in the orientation XOT measured were entirely caused by the head rotation. That is why we can interpret measured head orientation as head-to-trunk orientation. Its specified resolution is 0.235° at the acquisition rate of 100 Hz. In addition, simultaneous 3D measurements with a 3D laser scanner (LS) with a higher resolution compared to the HHC (one standard deviation after the calibration was 0.3 mm) were carried out. However, it is important to note, that the LS is inappropriate for *in-vivo* measurements, due to the long data acquisition times (about 5 s) and its lesser portability. With the presented experimental set-up we evaluated the impact of the apparatus’ resolution on the final–head rotation–precision.

A series of measurements of the fixed mannequin with a moveable head were acquired by all three measuring systems. The orientation of the head was measured in eight positions which imitated the standard positions for the determination of the cervical range of motion. These standard positions are:

1 Neutral head position, where the body planes of the head are parallel to the body planes of the trunk (this is also the reference position).

2 Natural head position, where the head is relaxed; in healthy subjects it should be the same as the reference one. In the case of the mannequin, the position was simulated as a random rotation around all axes.

3 Position of the maximal angle *φ* (flexion).

4 Position of the minimal angle *φ* (extension).

5 Position of the maximal angle *θ* (right rotation).

6 Position of the minimal angle *θ* (left rotation).

7 Position of the maximal angle *ψ* (left lateral tilt).

8 Position of the minimal angle *ψ* (right lateral tilt).

**Figure 6:**
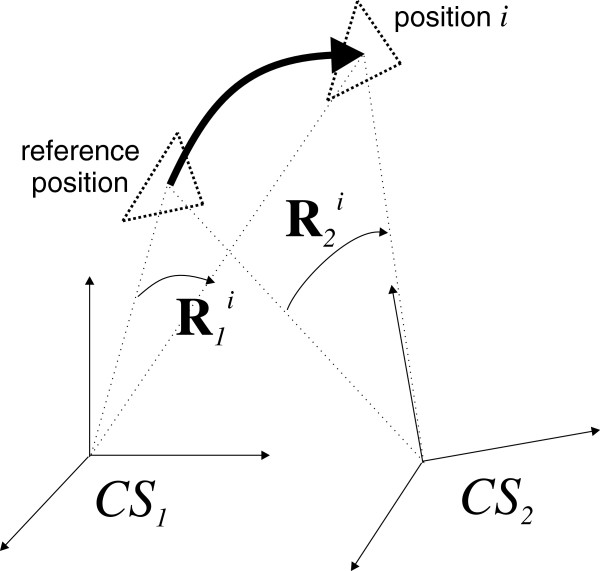
**Sketch of the conversion.** Sketch of the conversion from one coordinate system to another.

Since the coordinate systems of the utilized measuring instruments (*CS*_1_ and *CS*_2_ on Figure 6) are misaligned, the rotation matrix **R**_*g*_ between *CS*_1_ and *CS*_2_ has to be determined in order to measure the rotation angles about the same axes and be able to compare the rotation values obtained by different measuring instruments. If the first instrument measures the rotation of the object as **R**_1_, the second as **R**_2_ and **R**_1→2_ is the rotation **R**_1_ transformed to the *CS*_2_, the **R**_*g*_ can be calculated using the following procedure:

First, the rotation matrices are transformed into the axis-angle representation [19], where the rotation angle *α* is:

(5) $$\alpha =\text{arccos}\left(\frac{R_{1,1}+R_{2,2}+R_{3,3}-1}{2}\right)$$

The vector **k** which represents the rotation axis is:

(6) $$k=\frac{1}{2\sin\alpha }\left[\begin{array}{c} R_{3,2}-R_{2,3}\\ R_{1,3}-R_{3,1}\\ R_{2,1}-R_{1,2} \end{array}\right]$$

where *R*_*i*,*j*_ is the element of a rotation matrix in the *i*-th row and the *j*-th column. In this way, we calculate *α*_1_, *α*_2_, **k**_1_ and **k**_2_ from the known rotation matrices **R**_1_ and **R**_2_. Further we calculate the rotation axis **k**_*g*_ and the angle *α*_*g*_ between *CS*_1_ and *CS*_2_ as follows:

(7) $$k_{g}=\frac{k_{1}\times k_{2}}{ǁk_{1}\times k_{2}ǁ}$$

(8) $$\alpha_{g}=\text{arccos}\left(\frac{k_{1}\cdot k_{2}}{ǁk_{1}ǁ\cdot ǁk_{2}ǁ}\right)$$

In general the rotation matrix **R** from the known angle *α* and axis **k** = (*k*_1_,*k*_2_,*k*_3_)^*T*^ is calculated as:

(9) $$R=\left[\begin{array}{ccc} k_{1}^{2}v+\cos\left(\alpha \right) & k_{1}k_{2}v-k_{3}\sin\left(\alpha \right) & k_{1}k_{3}v+k_{2}\sin\left(\alpha \right)\\ k_{1}k_{2}v+k_{3}\sin\left(\alpha \right) & k_{2}^{2}v+\cos\left(\alpha \right) & k_{2}k_{3}v-k_{1}\sin\left(\alpha \right)\\ k_{1}k_{3}v+k_{2}\sin\left(\alpha \right) & k_{2}k_{3}v+k_{1}\sin\left(\alpha \right) & k_{3}^{2}v+\cos\left(\alpha \right) \end{array}\right]$$

where *v* = 1 − cos(*α*).

Ideally the angles *α*_1_ and *α*_2_ should be the same if both instruments measure the same object movement. But due to the limited measurement uncertainty some variation occurs. Therefore, the measurements were taken in all standard positions and $R_{g}^{i}$ for each *i*-th position was calculated. After that, the average rotation matrix ${\bar{R}}_{g}$ was calculated by averaging the corresponding Euler angles. So to transform **R**_1_ to **R**_1→2_, we calculate **k**_1_ and *α*_1_ as shown in Equations (7) and (8). Vector **k**_1→2_ and angle *α*_1→2_ in *CS*_2_ are then defined as:

(10) $$k_{1\to 2}={\bar{R}}_{g}\cdot k_{1}$$

(11) $$\alpha_{1\to 2}=\alpha_{1}$$

Once **k**_1→2_ and *α*_1→2_ are obtained, we calculate **R**_1→2_ as shown in the Equation (9). In the case of ideal measuring instruments, **R**_1→2_ and **R**_2_ should be exactly the same.

The differences of measured angles between the reference instrument (XOT) and 3D measuring instruments (LS and HHC) were analysed using single factor analysis of variance (ANOVA). The factor was measuring instrument. We analysed the main angles of all standard positions with the exception of the reference one. That results into seven ANOVA analyses between three sets of data. Differences were considered significant at p < 0.05. Bonferroni corrected t-test was used to pair-wise analyse the differences XOT vs. LS and XOT vs. HHC. Here, differences were considered significant at p < 0.0250.

### *In-vivo* verification

All *in-vivo* measurements were performed using the HHC apparatus. The human subject (diagnosed with cervical dystonia, aged 60) was dressed in a white T-shirt and a white headband was used to prevent the hair obstructing the subject’s face and extending the measurable surface. During the measuring procedure, the subject was resting against a wall. He was asked to rotate his head left and right as far as possible. Then he stepped away from the wall, relaxed for one minute and repeated the procedure for 13 times.

The presented research has received approval by the National Medical Ethics Committee of the Republic of Slovenia, which assesses the compliance with the Helsinki Declaration. The approval (No.: 133/04/13) is dated on 8th May 2013.

### *In-vitro* vs. *in-vivo* comparison

*In-vitro* and *in-vivo* comparison was done on the basis of an experiment where the HHC apparatus was used to measure head-to-trunk orientation of the mannequin and a healthy male (aged 29), respectively. To assure the repeatable head positioning, a laser projector, firmly attached to the head (in the direction of axis –Z on Figure 4), was used for illuminating the markers on the opposite wall. In the case of rotation and flexion/extension laser beam projector and one marker for each position were used. In the case of lateral tilt we used laser plane projector and two markers for each position, placed on the opposite wall approximately four meters apart. The neutral, moderate left and right rotation, moderate left and right lateral tilt and moderate flexion and extension were measured for 15 times. Only the main angles of each position were considered, since only these angles were monitored using the laser projector. Absolute values of the main angles (*θ* for left and right position, *φ* for flexion and extension and *ψ* for left and right lateral tilt) were summed to eliminate the effect of the mismatch of the laser placements on the both heads. The ranges of the rotation for both subjects were analysed using ANOVA single factor analysis. The factor was measured subject. Differences were considered significant at p < 0.05.

## Results and discussion

The uncertainty of the proposed method was assessed by three experiments. *In-vitro* verification was performed using the mannequin to evaluate the uncertainty of the method by comparing the results with the reference instrument. *In-vivo* verification was performed in order to evaluate the human influence on the measurement’s uncertainty, and *in-vitro* vs. *in-vivo* comparison to prove the relevance of *in-vitro* results. The gathered data was analysed using standard statistics’ approaches; unless noted otherwise, the precision of the measurements is defined as one standard deviation.

**Figure 7:**
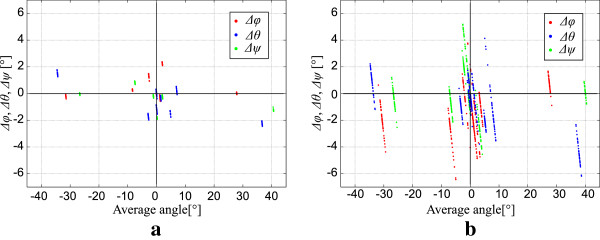
**Average rotations and differences.** The average rotation and differences of all standard positions, measured with LC **(a)** and HHC **(b)** compared to the reference XOT instrument.

The results of the *in-vitro* mannequin measurement are shown in Table 1. The reference instrument was the XOT, which recorded the data at each head position for one minute. 3D measurements were repeated twenty times using LS and forty times using HHC. The average Euler angles and standard deviations are shown for each instrument and for each head position. From these results we see that the XOT and LS have approximately the same precision (around 0.12°). This means that the proposed method of the head orientation extraction from the 3D shape works well. Additionally, we noticed that the HHC has somehow a lower precision (around 1.6°), which is attributed to the lower 3D measuring precision. The uncertainty of the calculated Euler angles compared to the XOT is presented by the Bland-Altman plots in Figure 7. The angle differences *Δφ*, *Δθ* and *Δψ* are defined as:

(12) $$\Delta \varphi =\varphi_{\text{XOT}}-\varphi_{3D}$$

(13) $$\Delta \theta =\theta_{\text{XOT}}-\theta_{3D}$$

(14) $$\Delta \psi =\psi_{\text{XOT}}-\psi_{3D}$$

**Table 1:** ***In-vitro *measured head rotation angles with XOT, LS and HHC**

**Position**			**XOT**	**LS**	**HHC**
			**mean ± std [°]**	**mean ± std [°]**	**mean ± std [°]**
Reference		*φ*	0.00 ± 0.12	0.01 ± 0.06	2.75 ± 0.98
		*θ*	0.00 ± 0.12	0.00 ± 0.05	−1.01 ± 0.56
		*ψ*	0.00 ± 0.13	0.00 ± 0.14	0.52 ± 0.37
Natural		*φ*	−8.23 ± 0.11	−8.47 ± 0.05	−5.47 ± 1.09
		*θ*	−26.68 ± 0.12	−26.63 ± 0.06	−26.14 ± 0.82
		*ψ*	1.31 ± 0.13	1.62 ± 0.13	1.45 ± 1.24
Rotation	left	*φ*	1.03 ± 0.11	1.37 ± 0.14	1.60 ± 1.92
		*θ*	−50.82 ± 0.12	−49.79 ± 0.12	−50.43 ± 1.90
		*ψ*	7.12 ± 0.12	6.91 ± 0.18	7.91 ± 1.92
	right	*φ*	−2.02 ± 0.11	−3.31 ± 0.16	−1.69 ± 1.31
		*θ*	40.00 ± 0.11	41.25 ± 0.07	39.88 ± 0.43
		*ψ*	−0.53 ± 0.13	0.66 ± 0.21	−0.07 ± 0.69
Lateral tilting	left	*φ*	3.13 ± 0.11	0.87 ± 0.09	4.06 ± 0.58
		*θ*	−1.19 ± 0.11	−0.99 ± 0.06	−3.16 ± 0.33
		*ψ*	35.55 ± 0.14	37.81 ± 0.11	39.81 ± 0.85
	right	*φ*	−0.04 ± 0.11	−0.02 ± 0.11	2.11 ± 1.05
		*θ*	1.93 ± 0.11	2.13 ± 0.10	4.60 ± 1.06
		*ψ*	−33.65 ± 0.12	−35.14 ± 0.14	−34.53 ± 0.88
flexion/extension	flexion	*φ*	27.87 ± 0.11	27.85 ± 0.06	27.10 ± 0.53
		*θ*	−0.73 ± 0.10	1.07 ± 0.06	0.04 ± 0.39
		*ψ*	4.08 ± 0.14	5.59 ± 0.16	6.33 ± 0.36
	extension	*φ*	−31.58 ± 0.13	−31.36 ± 0.09	−29.56 ± 1.01
		*θ*	−7.09 ± 0.12	−7.88 ± 0.06	−6.41 ± 0.69
		*ψ*	−3.7 ± 0.16	−2.02 ± 0.14	−2.69 ± 0.54

where angles with the index *XOT* were measured with XOT and angles with the index *3D* were measured with LS in Figure 7a and HHC in Figure 7b. The average angles of the position were determined as the average of the angles measured with XOT for each position.

Figure 7 indicates that the angle differences measured by LS are approximately half of the ones measured by HHC. This is a consequence of higher resolution of LS relative to HHC as they both use the same 3D surface reconstruction method and algorithm for the head orientation characterization. We expected less accurate results at a higher rotation of the head around the Y axis, due to the smaller area of overlapping between the reference and the current head surface. A healthy person can rotate the head up to 80° left or right [21]. In Table 1 we see that the standard deviation of the measured angles is indeed related to its amplitude. The left rotation (see Table 1, row “left rotation”, column “HHC”) has an amplitude of −50° and a standard deviation of 1.9° in case of using the HHC apparatus. On the other side, the right rotation has an amplitude of 40° and a standard deviation of 0.8° (see Table 1, row “right rotation”, column “HHC”), which confirmed our expectations. This effect can be minimized by adjusting the position of the measuring system in a way, that the impact angles between the optical axis of the measuring system and the surface of the head and trunk are as close as possible; if the head is rotated by 80° in relation to the trunk, the measuring system has to be placed in the direction rotated by 40° in relation to the trunk. The same problem can occur during flexion and extension, but those angles are usually smaller, up to 60° [21]. The least problematic is lateral tilt. Even in the case of large tilting angles, the impact angle between the measured surface and the optical axis of the measuring system does not change much. The problem of reduced overlapping is also insignificant.

The ANOVA analysis of the differences between main measured angles for all measuring instruments showed, that differences were significant at all positions. The critical F(2,57) was 3.1. Calculated F values were 6.7, 10.0, 271.1, 430.6, 35.2, 33.0, 74.6 for natural position, left rotation, right rotation, left lateral tilt, right lateral tilt, flexion and extension, respectively. Bonferroni corrected t-tests also showed that all differences were significant, with the exception of the natural position between XOT and LS (p = 0.12), left rotation between XOT and HHC (p = 0.56), right rotation between XOT and HHC (p = 0.11) and flexion between XOT and LS (p = 0.14). We think that the differences were caused by the non-ideal alignment between CS of the both measuring instruments and by the limited measuring accuracy.

The average angles and standard deviations of the 13 *in-vivo* measurements are seen in the Table 2. The standard deviation of the measured angles is increased compared to the results of the mannequin analysis. However, it is important to note that this deviation also includes scatter caused by the subject’s nervousness, misinterpretation of the command, tiredness, deformations of his face during head movement, measurement noise caused by the skin, etc.

**Table 2:** **Euler angles of the *in-vivo *measured head positions**

**Position**			**HHC**
			**mean ± std [°]**
Rotation	left	*φ*	−3.46 ± 1.75
		*θ*	−33.57 ± 2.64
		*ψ*	8.76 ± 2.61
	right	*φ*	−4.02 ± 1.67
		*θ*	32.26 ± 2.77
		*ψ*	−2.97 ± 2.93

The number of repeated measurements is 13.

The results of the *in-vitro* and *in-vivo* measurements are shown in Table 3. The critical F(1,28) for the statistical analysis was 4.2. From Table 3, column *ANOVA*, we see that there is no statistically significant difference between measured angles *θ* for the rotation of the human subject and the mannequin (F = 3.1). The difference between measured angles *φ* for the flexion/extension is marginally significant (F = 4.2). And the difference between measured angles *ψ* for the lateral tilt is statistically significant (F = 20.7). We explain this significance as the result of the limited human ability of accurate positioning. The illumination of the markers proved to be quite challenging task for the human subject. Tilting the head for the required angle was particularly difficult, since the subject had to ensure the overlapping of the laser line with two markers.

**Table 3:** ***In-vivo *and *in-vitro *measured angles**

**Range of**	**Angle**	**Mannequin**	**Human subject**	**ANOVA**
		**mean ± std [°]**	**mean ± std [°]**	**F value/critical F**
Rotation	*θ*	51.48 ± 0.49	51.94 ± 0.89	3.1/4.2
Lateral tilt	*ψ*	28.47 ± 0.73	29.05 ± 0.80	4.2/4.2
flexion/extension	*φ*	40.67 ± 0.35	41.43 ± 0.88	20.7/4.2

The number of repeated measurements is 15.

The proposed method enables a satisfactory level of measurement precision (2° for *in-vitro* and 3° for *in-vivo* measurement). It is a bit lower compared to the methods, presented in the Background section (from 0.15° to 2.7°) [2-9], but it has other advantages, like compensation of the whole body rotations, higher system portability, and finally no need for any device attached to the patient’s body.

Since the daily clinical practice needs a short, simple and precise tool for rating the improvement or deterioration of the cervical dystonia [22,23], we believe that the presented method has great potential to replace the currently used measuring systems.

## Conclusions

The new non-contact method for measuring head-to-trunk orientation is based on 3D surface acquisition. It enables the compensation of the movement of the measuring instrument or the subject’s body as a whole, is non-invasive, requires little additional equipment and causes little stress for the subject and operator. A subject is measured using the hand-held 3D apparatus which is based on the fringe projection technique. The apparatus is composed of a commercial DSLR camera and custom projection optics, which uses the light from the camera’s built-in flash. It is also highly portable and very user-friendly. The algorithm of the head-to-trunk orientation extraction is based on the separate alignment of the trunk and head on the reference measurement. The final results are the angles of rotation around each coordinate axis. Evaluation of the proposed method shows that the *in-vitro* measuring uncertainty is no more than 2° and *in-vivo* accuracy is less than 3°. The compassion of *in-vitro* vs. *in-vivo* measurements showed, that only the difference between flexion/extension measured angles was statistically significant. The differences between means were less than 1° for all ranges. The main contribution factors to the measurement’s uncertainty are the accuracy of the 3D apparatus and the subject’s time-varying deformation uncertainty. The measuring uncertainty is acceptable for the clinical use of the system, which can greatly improve the assessment of cervical dystonia in daily clinical practice.

## Abbreviations

FTP: Fourier transform profilometry; DSLR: Digital single-lens reflex; GPrS: Grating projection system; HHC: Hand-held 3D camera; LS: Laser scanner; XOT: Xsens 3DOF orientation tracker; GMS: Geomagic studio; CS: Coordinate system; ANOVA: Analysis of variance.

## Competing interests

The authors declare that they have no competing interests.

## Authors’ contributions

UP carried out the measurements, analysis, and drafted the manuscript. JD concepted and designed the experiment and revised the manuscript critically. JM proposed and conceived the research. MJ wrote the manuscript and designed the experimental system. All authors have read and approved the final version of the manuscript.
